# Haematological stress parameters and behavioural characteristics of dairy type goat kids compared to indigenous breeds during an intensive fattening programme

**DOI:** 10.5194/aab-63-441-2020

**Published:** 2020-12-02

**Authors:** Elif Ergul Ekiz, Hulya Yalcintan, Bulent Ekiz

**Affiliations:** 1 Department of Veterinary Physiology, Istanbul University-Cerrahpasa, Istanbul, 34500, Turkey; 2 Department of Animal Breeding and Husbandry, Istanbul University-Cerrahpasa, Istanbul, 34500, Turkey

## Abstract

In any production model, the extent to which the animals cope with the environment is important in terms of animal welfare and sustainability of production. The aim of the study was to investigate certain haematological parameters and behaviours of goat kids from dairy type Saanen and Maltese breeds via comparison with indigenous Hair and Gokceada breeds during the 10-week intensive fattening period. Eleven male goat kids each of Saanen, Maltese, Hair and Gokceada breeds were weaned at 3–3.5 months of age and then placed into four fattening pens prepared for each breed, separately. Cortisol, glucose and total protein levels were higher in Gokceada kids in the last period of the fattening compared to the kids of other breeds (P<0.05). In Gokceada kids, an evident decrease in the time spent hay feeding and on rumination and an increase in lying, standing and self-grooming behaviours were determined during the last 3 weeks of fattening. Moreover, there was a significant decrease regarding hay feeding in Hair goat kids in the last 2 weeks (P<0.05). Hair goat kids also exhibited less rumination behaviour compared to Saanen kids during the last 4 weeks of fattening (P<0.05). On the other hand, kids of dairy breeds did not express behavioural or biochemical stress responses during the fattening period. As a conclusion, when evaluated in terms of animal welfare, results of the current study may indicate that indigenous breeds, especially Gokceada kids, are not appropriate for intensive fattening in a pen.

## 1 Introduction

Approximately 99 % of the goat population in Turkey is composed of indigenous breeds such as Hair, Angora, Gokceada and Kilis. In the widely applied production system, these indigenous breeds are known for their low growth performance, low milk yield and small body size. On the other hand, these breeds have well adapted to survive in harsh environmental conditions and in poor feeding (Özcan et al., 2010).

In recent years, with the increasing demand for goat milk and goat milk products such as ice cream and cheese, many dairy goat enterprises under intensive or semi-intensive conditions have been established. In these farms, Saanen and Maltese breeds, which are generally known for their high milk yield, are preferred (Ekiz et al., 2010; Yalcintan et al., 2018). Milk constitutes the main income item in dairy goat farms, and the sale of male kids is also an important subsidiary income. In order to obtain as much saleable milk as possible from their mothers, goat kids are mostly weaned at an early age and referred to slaughter without any fattening. Therefore, low weight carcasses are obtained from these male kids due to their low live weight at slaughter. On the other hand, low-weight carcasses are also obtained in the extensive production system carried out with indigenous breeds, and the meat production potential of the kids is not sufficiently utilized (Ekiz et al., 2010; Özcan et al., 2010; Yilmaz et al., 2010). In both production models made with indigenous breeds under the extensive system and with dairy breeds under the semi-intensive or intensive systems, fattening of kids in the intensive fattening programme after weaning can be considered as an effective method to increase the production of kid meat (Yalcintan et al., 2018).

In the last decades in Western society, consumers are not only concerned with the price of products and the safety of their food, but also with how their food is produced and especially with how animals are raised, transported and slaughtered (Éloit, 2017). Also, in a study conducted with Turkish consumers, great support about welfare-friendly animal production was observed (İzmirli and Yaşar, 2010). Animal welfare is one of the elements of sustainability of animal production (Gamborg and Sandøe, 2005). Therefore, when deciding on the production system to be applied, it should be taken into consideration whether the animals to be used are able to cope with the environment.

The aim of the current study was to evaluate the intensive fattening programme applied after weaning in order to produce more meat from goat kids in four different breeds with the perspective of animal welfare. In this context, certain behaviours and haematological parameters that may be indicators of stress in the goat kids from dairy type Saanen and Maltese breeds via comparison with indigenous Hair and Gokceada breeds were investigated during the 10-week intensive fattening period.

## 2 Materials and methods

The experimental procedures of the study were approved by the Ethic Committee of Istanbul University (approval no. 53/09).

### 2.1 Animals and management

The study was carried out in the farm animals unit of the Veterinary Faculty Clinic at Istanbul University, between mid-April and the end of June. Eleven male goat kids from each of Saanen, Maltese, Hair and Gokceada breeds were purchased after weaning from the same commercial farm. Goat kids were kept with their mothers for the first month after birth. In the following months, the mothers were taken to the pasture in the morning, and in the evening they stayed with the kids in the shelter. Kids had also free access to commercial grower concentrated feed and hay. The kids suckled their mothers until they were 3–3.5 months old at the commercial farm where they were born.

All kids were weaned on the same day (at 3–3.5 months), and the next day they were transported to veterinary faculty with the same vehicle. In the farm animals unit, four pens (each of 3.4×3.9 m; 13 $m^{2}$) were prepared in the same corridor. A feeder (3.9×0.7 m) for concentrate feed was placed adjacent to the long side of the fattening pen, and a feeder (3×1 m) for hay was placed adjacent to the short side of the pen. The water container was placed adjacent to the other side wall of the fattening pen. The pens were cleaned and equipped with straw as bedding material before the kids arrived. Kids from the same breeds were placed into the same fattening pen.

In order to supply free access to feed, commercial concentrated feed (16.9 % CP and 2820 kcalkg-1 ME) was given to kids twice a day at 08:30 and 16:00 by considering their consumptions. Kids also received free access to alfalfa hay (7.34 % CP and 2050 kcalkg-1 ME) and clean/fresh water during the fattening period. The daily mean (min–max) temperature was 11.6 ${}^{\circ }$C (7.4–15.8 ${}^{\circ }$C) in April, 16.1 ${}^{\circ }$C (11.6–20.6 ${}^{\circ }$C) in May, and 20.5 ${}^{\circ }$C (15.8–25.2 ${}^{\circ }$C) in June.

### 2.2 Behavioural observations

In the study, direct observations for certain individual, feeding and abnormal behaviours were performed to observe behavioural changes of goat kids in different periods of fattening and to identify possible behavioural differences among breeds. During the 10-week fattening period, each group was observed 2 days a week and twice on each observation day (between 09:30–11:30 in the morning, between 14:00–16:00 in the afternoon). The behavioural observations were performed by the two experienced observers 1 m away from the pens. Specific numbers were painted on the hip regions and on the back of the animals so that all the kids could be identified individually by the observers from a distance. In order to eliminate the possible observer effect, observation of a breed group was made rotationally by two observers. Observers took their places at the observation point 15 minutes before the start of observation period to ensure that the kids got used to the observer.

Time-sampling observation method (Bogner, 1984; Mitlöhner et al., 2001) was used during the observation of lying, standing, walking, concentrate feeding, hay feeding, drinking, and rumination behaviours. Animals exhibiting these behaviours were recorded at the end of each 5 min on the previously prepared charts. On the other hand, since self-grooming, butting other animals and abnormal oral activities (licking or gnawing of paddock equipment such as wall, fence and manger) were expressed more rarely by animals, these behaviours were recorded at the time they were observed. Descriptions of the behavioural characteristics investigated in the study were presented in Table 1 (De et al., 2019; Tölü and Savaş, 2007; Tölü et al., 2016; Ugur et al., 2004).

During the data-editing processes, data of each animal regarding lying, standing, walking, concentrate feeding, hay feeding, drinking and rumination behaviours for each observation period were converted to percentage values, which give the proportion of each behavioural activity within the total frequency of these behaviours. As a result of this process, a total of 40 (10weeks×2 d in a week ×2 observation period in a day) data for each of the above-mentioned behaviours were obtained for each animal. On the other hand, behavioural activities, recorded as they were observed, were arranged as the frequency of these behaviours displayed by each animal in every 2 h observation period.

### 2.3 Blood sampling and analyses

Blood samples were taken from the jugular vein by the same trained person at week 2, 4, 6 and 8. To make it easier to draw blood, necks of kids were shaved the day before sampling. Blood samples were taken at 09:30 on days without behavioural observation. To avoid excessive stress, blood sampling of each kid was completed in about 1 min.

Two blood samples (EDTA and heparinized) were taken from each goat kid at each blood sampling. EDTA blood samples were used to determine the packed cell volume via standard capillary microhaematocrit method. The heparinized blood samples were centrifuged (3500 rpm for 15 min) within 1 h after blood collection, and the plasma samples obtained were kept at -85 ${}^{\circ }$C until analysis.

The commercial diagnostic ELISA kit (DiaMetra, Foligno, Italy; ref. DK0001; lot no. 2186) was used for measurement of plasma cortisol concentration. The assay sensitivity was 5 $\mathrm{ng}\;{\mathrm{mL}}^{-1}$, whereas intra- and inter-assay variations were 7 and 9.32 %. A multiparametric auto-analyser (TMS 1024, TokyoBoeki Medical System, Tokyo, Japan) and its accompanying commercial kits (Spinreact, Girona, Spain) were used in the determination of plasma glucose (ref. 1001192; lot no. 172), total protein (ref. 1001291; lot no. D195), CK (ref. 1001050; lot no. 2188:T) and LDH (ref. 1001260; lot no. 2216T) concentrations.

### 2.4 Statistical analysis

The Shapiro–Wilk test was used to check the normality of data. In the first step of the statistical analyses of the haematological parameters (glucose, LDH, CK, total protein, cortisol and PCV), repeated measures ANOVA was used. The statistical model of these analyses included breed as a “between-subject factor” and sampling week as a “within-subject factor”. In the second step, one-way ANOVA and Duncan's multiple range tests were applied to determine the effect of breed for each sampling week, separately. On the other hand, repeated measures ANOVA model, which included sampling week as a “within-subject factor”, was used to determine the effect of sampling week on haematological parameters for each bread, separately.

In the statistical analyses of lying, walking, concentrate feeding, hay feeding, rumination and self-grooming behaviour data, similar procedures with haematological data were applied, except the content of repeated measures ANOVA. Observation hours (morning or afternoon) were also included as a “within-subject factor” in the repeated measures ANOVA models for these behavioural characteristics.

Data of standing, drinking, butting other animals and abnormal oral activities did not fit in the normal distribution. Therefore, the Kruskal–Wallis test was used to compare breeds for data of these behaviours. In these cases, data of each observation week were analysed separately, and percentages or frequencies of these behaviours during the whole observation week were evaluated. The Friedman test was used to evaluate the change in different weeks within a breed.

**Table 1:** Description of individual, feeding and abnormal behaviours investigated in the study.

Behaviour	Description
Behaviours recorded at the end of each 5 min with the time sampling method	
Lying	Lying in a resting position without showing rumination or any other behaviour
Standing	Standing in a resting position without showing rumination or any other behaviour
Walking	The goat kid moves from one place to another
Concentrate feeding	Concentrate feed consumption
Hay feeding	Hay feed consumption
Drinking	Water consumption
Rumination	Ruminating either in lying or standing position
Behaviours recorded at the time they were observed	
Self-grooming	The kid grooms itself at any part of the body or legs
Abnormal oral activities*	Licking of paddock walls or fences; licking of the manger or water containers; gnawing of paddock walls or fences; gnawing of the manger or water containers
Butting other animals	Butting another kid's body part by using their head

${}^{*}$Licking of paddock walls or fences, licking of the manger or water containers, gnawing of paddock wall or fence, and gnawing of the manger or water containers were recorded separately during behavioural observations. During the data-editing phase, these behaviours were gathered under one title as abnormal oral activities.

The chi-squared test was applied to compare breeds in terms of mortality rate. The statistical analyses were performed using SPSS, version 13.0 (SPSS Inc., Chicago, IL, USA). Differences were considered significant if P<0.05.

## 3 Results

Plasma glucose levels in different periods of fattening due to breed are presented in Table 2. When the glucose data of four breeds in all weeks were considered, the effect of breed × week interaction was significant (P=0.023) in repeated measures ANOVA statistics. Breed had no significant influence on plasma glucose levels at weeks 2, 4 and 6 (P>0.05), while Gokceada kids had higher values than kids of other breeds at week 8 (P<0.01). On the other hand, as the fattening week progresses in Saanen, Maltese and Hair kids, a gradual decrease in plasma glucose level was determined (P<0.05), whereas in Gokceada kids there was no such trend (P>0.05).

**Table 2:** Plasma glucose, lactate dehydrogenase (LDH) and creatine kinase (CK) levels in different periods of fattening due to breed.

Parameters	Week	Saanen		Maltese		Hair		Gokceada		P value${}^{g}$
		Mean	SE	Mean	SE	Mean	SE	Mean	SE	
Glucose, $\mathrm{mg}\;{\mathrm{dl}}^{-1}$	2	72.00${}^{c}$	2.43	69.45${}^{c}$	2.22	68.56${}^{c}$	1.55	70.11	1.92	0.695
	4	70.82${}^{c}$	3.22	66.64${}^{\text{cd}}$	1.11	66.89${}^{c}$	1.74	70.56	1.11	0.327
	6	65.27${}^{d}$	2.36	63.55${}^{\text{de}}$	1.37	62.78${}^{\text{cd}}$	2.27	69.67	1.11	0.078
	8	58.36${}^{\text{b,e}}$	3.82	56.64${}^{\text{b,e}}$	2.72	58.56${}^{\text{b,d}}$	2.60	71.11${}^{a}$	1.10	0.005
	P value${}^{f}$	<0.001		<0.001		0.015		0.897		
LDH, $U\;l^{-1}$	2	887.54	71.62	839.55	60.03	941.00	96.27	923.00	89.05	0.803
	4	865.55	60.22	827.82	93.10	985.89	182.44	952.67	108.05	0.738
	6	896.00	42.21	982.82	54.96	938.22	29.76	897.11	68.58	0.562
	8	952.45	37.93	1052.18	47.45	1012.00	21.12	985.89	55.12	0.379
	P value${}^{f}$	0.415		0.063		0.805		0.735		
CK, $U\;l^{-1}$	2	355.64	83.22	327.09	25.76	274.33	39.29	312.22	27.02	0.749
	4	293.91	27.11	320.91	30.67	298.00	22.08	311.89	18.75	0.866
	6	333.27	36.62	353.82	52.07	294.67	30.32	298.22	16.48	0.652
	8	281.73	31.07	414.18	66.17	274.67	26.71	300.33	15.63	0.077
	P value${}^{f}$	0.540		0.295		0.844		0.925		

${}^{\text{a,b}}$ Means in the same row with different letters differ significantly (P<0.05).${}^{\text{c,d,e}}$ Means in the same column with different letters differ significantly (P<0.05).${}^{f}$ Significance level of differences between fattening weeks within breed according to results of repeated measures ANOVA statistics.${}^{g}$ Significance level of differences between breeds for the same row according to results of one-way ANOVA statistics.

Breed × week interaction had no significant influence on plasma LDH and CK concentrations (P>0.05) in repeated measures ANOVA statistics. In addition, the effect of breed on plasma LDH and CK concentrations was not significant in any blood collection week (Table 2).

In the second week, plasma total protein level was similar in Saanen, Maltese, Hair and Gokceada kids (P>0.05; Table 3). However, a gradual decrease in plasma total protein level was observed in Hair goat kids starting from the fourth week (P=0.011) and in Maltese kids after the sixth week (P=0.014). These changes led to the fact that Gokceada kids had a higher total protein levels in the fourth and eighth weeks compared to Hair goat kids (P<0.01 and P<0.05, respectively) and in the sixth week compared to the kids from all other breeds (P<0.05).

In blood samples taken at the second week of fattening, Saanen kids had higher cortisol concentrations compared to Maltese and Hair goat kids (P=0.007; Table 3). The effect of breed on plasma cortisol level was not significant at fourth and sixth weeks (P>0.05). At the eighth week, Gokceada kids had higher plasma cortisol concentration than the kids of other goat breeds (P=0.011). On the other hand, the effect of blood sampling week on cortisol level was not significant in any breed (P>0.05). In terms of PCV, the differences among breeds were not significant in any period of fattening (P>0.05; Table 3). On the other hand, as the fattening week progressed, a gradual decrease in PCV was determined in all breeds (P<0.05).

**Table 3:** Packed cell volume (PCV) and plasma cortisol and total protein levels in different periods of fattening due to breed.

Parameters	Week	Saanen		Maltese		Hair		Gokceada		P value${}^{h}$
		Mean	SE	Mean	SE	Mean	SE	Mean	SE	
Total protein, $g\;{\mathrm{dl}}^{-1}$	2	6.43	0.11	6.46${}^{d}$	0.10	6.28${}^{d}$	0.10	6.64	0.11	0.155
	4	6.46${}^{a}$	0.11	6.61${}^{\text{a,d}}$	0.07	6.19${}^{\text{b,de}}$	0.06	6.67${}^{a}$	0.11	0.005
	6	6.22${}^{b}$	0.16	6.35${}^{\text{b,d}}$	0.14	6.14${}^{\text{b,de}}$	0.53	6.74${}^{a}$	0.11	0.018
	8	6.41${}^{\text{ab}}$	0.08	6.05${}^{\text{bc,e}}$	0.19	5.94${}^{\text{c,e}}$	0.14	6.48${}^{a}$	0.12	0.030
	P value${}^{g}$	0.433		0.014		0.011		0.239		
Cortisol, $\mathrm{ng}\;{\mathrm{mL}}^{-1}$	2	21.29${}^{a}$	2.94	10.14${}^{b}$	1.32	12.47${}^{b}$	2.89	16.60${}^{\text{ab}}$	1.72	0.007
	4	22.44	3.54	13.58	2.04	14.43	2.08	18.98	3.30	0.102
	6	15.39	2.42	14.55	1.85	14.26	1.15	20.74	1.43	0.081
	8	15.31${}^{b}$	1.48	14.67${}^{b}$	1.03	13.90${}^{b}$	2.01	21.13${}^{a}$	1.63	0.011
	P value${}^{g}$	0.089		0.101		0.841		0.130		
PCV, %	2	30.00${}^{d}$	1.33	30.09${}^{d}$	0.92	29.56${}^{d}$	0.77	30.22${}^{d}$	1.06	0.977
	4	26.64${}^{e}$	0.45	28.36${}^{d}$	0.98	27.44${}^{\text{de}}$	0.60	28.78${}^{\text{de}}$	1.04	0.245
	6	27.27${}^{\text{de}}$	0.95	28.00${}^{d}$	0.73	26.11${}^{\text{ef}}$	0.48	28.78${}^{\text{de}}$	0.94	0.170
	8	27.27${}^{\text{de}}$	0.49	26.36${}^{e}$	1.09	24.89${}^{f}$	0.65	26.78${}^{e}$	0.80	0.217
	P value${}^{g}$	0.011		0.011		<0.001		0.031		

${}^{\text{a,b,c}}$ Means in the same row with different letters differ significantly (P<0.05).${}^{\text{d,e,f}}$ Means in the same column with different letters differ significantly (P<0.05).${}^{g}$ Significance level of differences between fattening weeks within breed according to results of repeated measures ANOVA statistics.${}^{h}$ Significance level of differences between breeds for the same row according to results of one-way ANOVA statistics.

During the 10-week fattening period, two kids from both Gokceada and Hair goat breeds died due to asphyxia resulting from severe cellular and necrotic pneumonia (results are not shown in tables or figures). However, according to the result of chi-square analysis, there was no statistically significant difference among breeds in terms of mortality rate (chi-square =4.4; P=0.221).

**Figure 1:**
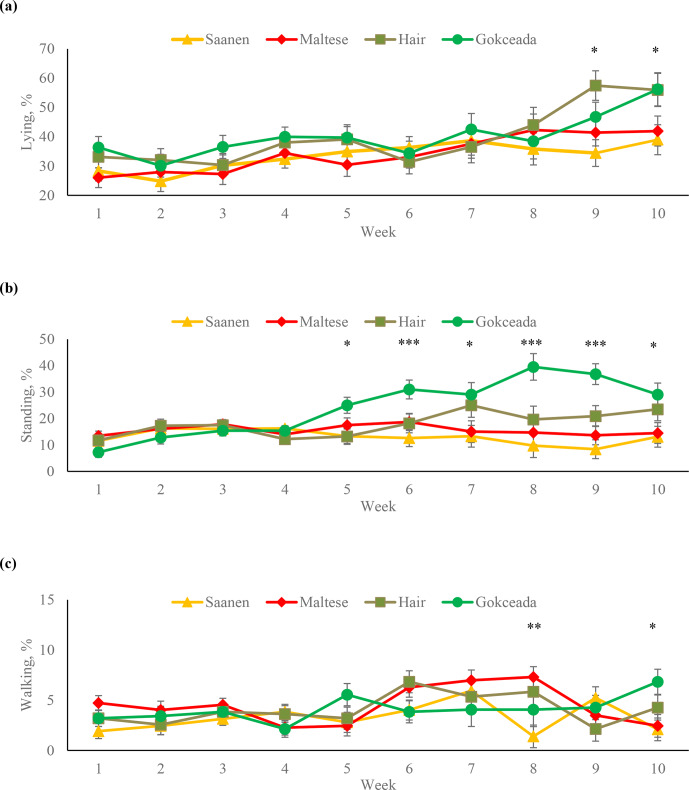
Percentages of **(a)** lying, **(b)** standing and **(c)** walking behaviours at different fattening weeks according to breed. ${}^{*}$ P<0.05. ${}^{**}$ P<0.01. ${}^{***}$ P<0.001.

The percentages of the time spent lying, standing and walking behaviours at different weeks are presented in Fig. 1. In the first 8 weeks, differences among breeds in terms of time spent lying were not significant (P>0.05). It was observed that the Hair goat kids in the 9th week and the Hair and Gokceada kids in the 10th week expressed more lying behaviour compared to the kids of dairy breeds (P<0.05). In the Hair goat and Gokceada breeds, the percentages of lying behaviour increased significantly in the last 2 weeks compared to the first 6 weeks (P<0.05). In terms of the time spent standing, from the fifth week to the end of the fattening, the kids of Gokceada breed had higher values than Saanen and Maltese breeds (P<0.05). Percentages of time spent walking in Saanen kids were lower than that of Maltese kids at the 8th week (P<0.01) and lower than that of Gokceada kids at the 10th week (P<0.05).

**Figure 2:**
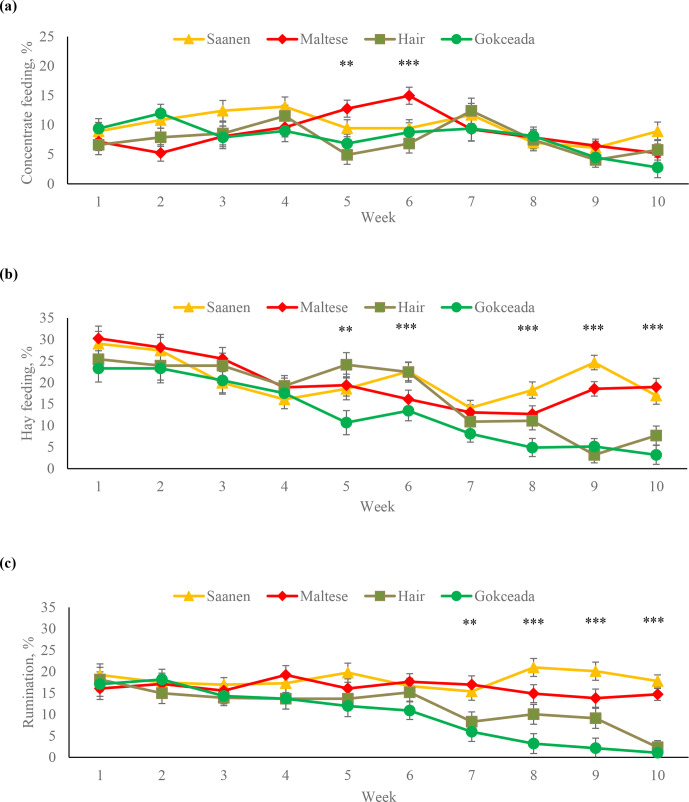
Percentages of **(a)** concentrate feeding, **(b)** hay feeding and **(c)** rumination behaviours at different fattening weeks according to breed. ${}^{**}$ P<0.01. ${}^{***}$ P<0.001.

The percentages of the time spent concentrate feeding, hay feeding and on rumination behaviours at different weeks are shown in Fig. 2. Maltese kids spent more time on concentrate feeding at fifth and sixth weeks compared to Hair goat and Gokceada kids (P<0.01). In terms of time spent on hay feeding, there were significant breed differences at weeks 5, 6, 8, 9 and 10. Gokceada kids spent less time hay feeding than kids of dairy breeds at 5th, 8th, 9th and 10th weeks (P<0.01). At fifth and sixth weeks, Gokceada kids also spent less time hay feeding than Hair kids (P<0.01). Regarding the percentages of expression of this behaviour at different weeks of fattening within a breed, there was a significant decrease in Hair goat kids in the last 2 weeks and in Gokceada kids in the last 4 weeks (P<0.05). In terms of percentage of time spent on rumination, there were significant differences among breeds in the last 4 weeks of the fattening period. Gokceada kids expressed less rumination behaviour compared to the dairy breeds during the last 4 weeks (P<0.01). In this period, Hair goat kids also exhibited less rumination behaviour compared to Saanen kids (P<0.05).

Overall mean values for the percentage of time spent drinking were 1.3 %, 1.6 %, 1.4 % and 1.2 % for Saanen, Maltese, Hair goat and Gokceada kids, respectively (results are not shown in tables or figures), and there were no significant differences among breeds at any week of the fattening period (P>0.05).

**Figure 3:**
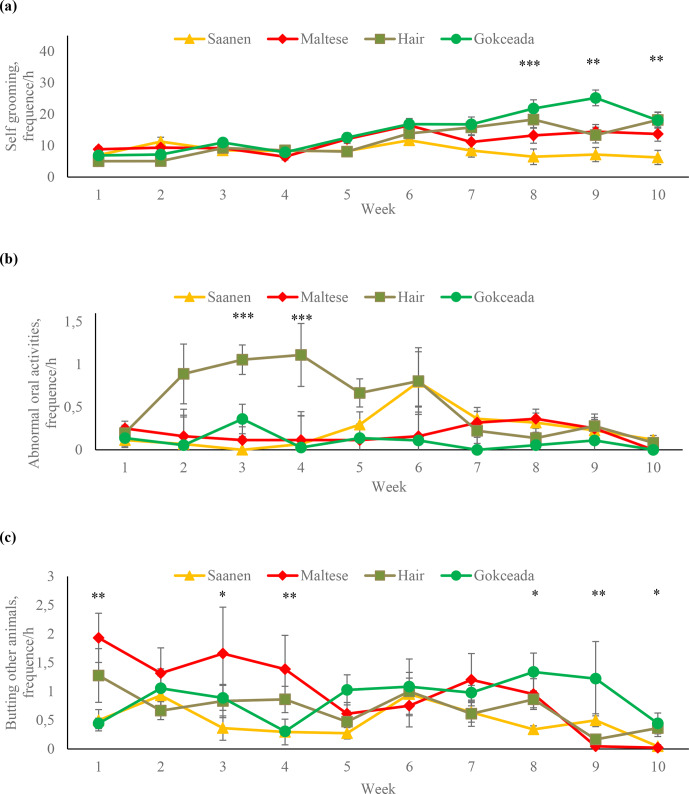
Frequencies of **(a)** self-grooming, **(b)** abnormal oral activities and **(c)** butting other animals behaviours at different fattening weeks according to breed. ${}^{**}$ P<0.01. ${}^{***}$ P<0.001.

The frequencies of self-grooming, abnormal oral activities and butting other animals behaviours at different weeks are presented in Fig. 3. The effect of fattening week on frequency of self-grooming behaviour was not significant in Saanen, Maltese and Hair goat kids during the whole fattening period. However, in Gokceada kids, there was a significant increase in the frequency of self-grooming behaviour during the last 3 weeks of fattening. As a result of these changes, Gokceada kids expressed more self-grooming behaviour in the 8th week compared to the Saanen and Maltese kids, in the 9th week compared to kids from all other genotypes, and in the 10th week compared to the Saanen kids.

In terms of abnormal oral activities, statistically significant breed effect was found only in third and fourth weeks (Fig. 3b). In these weeks, Hair goat kids showed more abnormal oral activities compared to kids of other breeds (P<0.001). Moreover, in the third week, Gokceada kids displayed more abnormal oral activities than Saanen kids (P<0.01).

Maltese kids showed more “butting other animals” behaviour than Saanen and Gokceada kids in the first week and more than Saanen kids in the 3 and 4 weeks (Fig. 3c). An increase in the frequency of “butting other animals” behaviour was determined in Gokceada kids in the eighth and ninth weeks of the fattening. Gokceada kids expressed more “butting other animals” behaviour than Maltese kids in the last 2 weeks and Saanen kids in the 8th and 10th weeks.

## 4 Discussion

Fattening of farm animals under intensive feeding programme in a pen can cause chronic stress in animals due to a highly concentrated diet and/or boring environment (Aguayo-Ulloa et al., 2013; Keskin et al., 2004). When animals are exposed to distress or environmental challenges, the stress response is an important physiological way of self-protection and adaptation to new conditions (Miranda-de la Lama et al., 2013). Numerous environmental or individual factors may influence the stress responses of animals and therefore the degree of changes in physiological variables due to stressors. One of these factors is the breed or genotype of animal (Broom, 2005). In the current study, in the first blood samples, the cortisol level in Saanen kids was higher than in Maltese and Hair goat kids. This result may indicate that Saanen kids experienced more stress during the first period of fattening. However, plasma glucose results did not support this interpretation. On the other hand, plasma glucose and cortisol levels measured at eighth week of the fattening were higher in Gokceada kids than the other breeds. Moreover, at the eighth week of the fattening, total protein levels of Gokceada kids were also higher than those of Maltese and Hair goat kids. These results indicate that Gokceada kids experienced more stress in the eighth week compared to kids of other breeds. Supporting our results, significant breed differences were also observed in a previous study (Kadim et al., 2006), in which the stress responses of three Omani goat breeds to transport were evaluated. In that study (Kadim et al., 2006) Batina goats had higher cortisol concentrations than Jabal Akhdar and Dofari goats, and it was concluded that Batina goats may be more susceptible to stress compared to Jabal Akhdar and Dofari breeds. In a previous study (Banerjee et al., 2015) conducted to investigate the adaptability of Indian goat breeds to hot and cold climates, significant differences were found between heat-tolerant (Sirohi and Barbari) and cold-tolerant (Gaddi and Chegu) goat breeds for blood glucose and cortisol levels. The latter researchers (Banerjee et al., 2015) noted that the adaptation processes to heat and cold were different among breeds. Horton and Burgher (1992) determined that the cortisol levels of St. Croix, Barbados, Dorset and Katahdin lambs were similar during the first 14 d of the 84 d feeding programme, but latter cortisol levels were higher in the Katahdin lambs compared to other breeds. In that study, higher cortisol levels in Katahdin lambs were attributed to the higher weight gain and more efficient feed utilization (Horton and Burgher, 1992). Significant breed differences in terms of cortisol response to the stress were also found by Hall et al. (1998) for sheep genotypes, and the researchers concluded that the increase in cortisol level due to stress was higher in highland sheep than their lowland counterparts.

In the current study, total protein level of Gokceada kids was higher in blood samples taken in the eighth week compared to Maltese and Hair goat kids. This result was compatible with the results of cortisol and glucose. Moreover, total protein level of Gokceada kids was also higher than those of the other breeds at sixth week. An increase in blood total protein level due to various stress factors in goats and sheep has also been reported previously (Bórnez et al., 2009; Kadim et al., 2006; Ribeiro et al., 2018). Although cortisol, glucose and total protein levels were higher in Gokceada kids in the last period of fattening compared to other breeds, the levels of these variables were within the ranges reported for unstressed goats kept in holding pens or for the period before stress application for various breeds (Alcalde et al., 2017; Jones and Allison, 2007; Kadim et al., 2006; Kannan et al., 2003).

CK and LDH are biochemical indicators used to examine possible trauma, vigorous exercise or tissue damage. An increase in plasma CK concentration is observed if muscle damage occurs or high physical activity exists. The increase in plasma LDH level is mainly caused by tissue damage (Bórnez et al., 2009; De la Fuente et al., 2010; Kannan et al., 2003; Tadich et al., 2009). In the current study, the fact that both the fattening week and breed had no effect on plasma CK and LDH suggests that the animals were not exposed to strenuous muscle activity during the fattening period. Moreover, kids from Saanen, Maltese, Hair goat and Gokceada breeds had similar PCV values throughout the fattening period. PCV values obtained in the study were also within the normal reference ranges (22 %–38 %) for healthy goats (Jones and Allison, 2007).

Since farm animals express their internal states with behaviours, determination of behavioural changes in an animal points out the changes in the animal's state. Behavioural changes in farm animals may be the result of various challenges, such as the inability to express normal behaviour, which is caused by the farm environment, disease or injury (Matthews et al., 2017). Therefore, the assessment of behavioural changes is a widely used non-invasive method to measure animal welfare at farm level (Aguayo-Ulloa et al., 2013).

Depending on the breed, goat kids spent 33.5 %–40.1 % of their time lying, and this behaviour was the most commonly exhibited behaviour in the time budget throughout the study. A similar result was also reported by Bøe et al. (2007) for dairy goats and Aguayo-Ulloa et al. (2013) for lambs. When the weekly changes in lying behaviour for each breed were examined, it is noteworthy that while the percentage of lying in the dairy breeds throughout the whole study were stable, indigenous Hair goat and Gokceada kids increased their time spent lying in the last 2 weeks of the fattening period. An increase in standing behaviour was also observed in Gokceada kids after the fifth week. On the other hand, time spent hay feeding decreased in Hair goat kids in the last 2 weeks and in Gokceada kids in the last 4 weeks. Also, there was a significant decrease in rumination behaviour in Gokceada kids after the sixth week and in Hair goat kids in the last week. These results indicate that there was a significant change in the shares of behaviours in the time budget in the kids of indigenous breeds towards the end of the fattening and especially in the last 2 weeks. These changes were much more evident in Gokceada kids. These behavioural differences among breeds were also in accordance with the results seen in the cortisol and glucose levels. Significant breed differences in terms of lying, standing and feeding behaviours were also reported in a previous study conducted with indigenous goat breeds of Graúna, Blue and Moxotó kept in the same environment (Silva et al., 2014). Behavioural differences among genotypes might be explained by the fact that genotypes may differ in their responses to the combination of stressors and also their degree of habituation to stresses (Hall et al., 1998).

In the current study, when the weekly changes in self-grooming behaviour were examined for each breed; the frequency of this behaviour was observed to be stationary in Hair goat, Saanen and Maltese kids throughout the study. However, significant increases were observed in Gokceada kids during the last 3 weeks of the fattening. Moreover, the frequency of “butting other animals” behaviour in the last 3 weeks of fattening was also higher in Gokceada kids. These results were in accordance with the changes in individual and feeding behaviours in different weeks of fattening and also with the results of biochemical stress response variables. On the other hand, Hair goat kids showed more “abnormal oral activities” than other breeds at the third and fourth weeks of fattening. Gokceada goats have been reared on the island of Gokceada for centuries in natural pastures under conditions close to those in the wild. Gokceada goats have the opportunity to graze freely on the island all year-around and have adapted to survive in poor feeding and environmental conditions by natural selection (Ekiz et al., 2010). The indigenous Hair goat breed, which constitutes approximately 96 % of the total goat population in Turkey, is usually reared in forested and mountainous areas under an extensive system (Deger Oral Toplu and Altınel, 2008). Therefore, it can be said that the environmental conditions within the fattening pen were quite barren for indigenous breeds compared to their natural environment. This may be an explanation for the kids of indigenous breeds being less successful in adapting to the intensive fattening in a pen than the dairy breeds raised generally in semi-intensive or intensive conditions.

When animals are re-grouped or unfamiliar individuals are placed in a pen, inevitably agonistic behaviours are observed for the establishment and maintenance of dominance relationships (Miranda-de la Lama and Mattiello, 2010). Aggressive interactions between animals may also increase where resources are limited, such as limited space for the animal to move freely (Andersen and Bøe, 2007). In Maltese kids, higher frequencies of “butting other animals” behaviour in the initial weeks of fattening might be attributed to the struggle of animals to constitute an in-group dominance hierarchy. On the other hand, current results show that frequencies of butting other animals were lower than those reported for several goat breeds previously (Bøe et al., 2012; Tölü et al., 2016).

## 5 Conclusions

In the conditions of the current study, kids of dairy breeds did not express behavioural or biochemical stress responses during the fattening period. On the other hand, results regarding biochemical stress response variables and behaviours may suggest that, especially in the last 2 weeks of the fattening, Gokceada kids were less successful in attempting to cope with the confined environment that they live in. Therefore, it might be concluded that, indigenous breeds, especially Gokceada kids, are not appropriate for intensive fattening in a pen. Standard error values obtained for some behavioural traits in the study were slightly high. This might be a result of an insufficient number of animals in each breed and, therefore, might be considered as a limitation of the study. Further research is needed to investigate the fattening performance beside welfare evaluation of goat kids from indigenous and dairy breeds.

## Data Availability

The data are available upon request from the corresponding author.
